# Large-scale transcriptomic analysis of coding and non-coding pathological biomarkers, associated with the tumor immune microenvironment of thyroid cancer and potential target therapy exploration

**DOI:** 10.3389/fcell.2022.923503

**Published:** 2022-08-03

**Authors:** Ming-Lang Shih, Bashir Lawal, Sheng-Yao Cheng, Janet O. Olugbodi, Ahmad O Babalghith, Ching-Liang Ho, Simona Cavalu, Gaber El-Saber Batiha, Sarah Albogami, Saqer S. Alotaibi, Jih-Chin Lee, Alexander T. H. Wu

**Affiliations:** ^1^ Division of General Surgery, Department of Surgery, Tri-Service General Hospital, National Defense Medical Center, Taipei, Taiwan; ^2^ PhD Program for Cancer Molecular Biology and Drug Discovery, College of Medical Science and Technology, Taipei Medical University and Academia Sinica, Taipei, Taiwan; ^3^ Graduate Institute for Cancer Biology and Drug Discovery, College of Medical Science and Technology, Taipei Medical University, Taipei, Taiwan; ^4^ Department of Otolaryngology-Head and Neck Surgery, Tri-Service General Hospital, National Defense Medical Center, Taipei, Taiwan; ^5^ Department of Biochemistry, Bingham University, Karu, Nigeria; ^6^ Medical Genetics Department, Faculty of Medicine, Umm al-Qura Univeristy, Mecca, Saudi Arabia; ^7^ Division of Hematology and Oncology Medicine, Department of Internal Medicine, Tri-Service General Hospital, National Defense Medical Center, Taipei, Taiwan; ^8^ Faculty of Medicine and Pharmacy, University of Oradea, Oradea, Romania; ^9^ Department of Pharmacology and Therapeutics, Faculty of Veterinary Medicine, Damanhour University, Damanhour, Egypt; ^10^ Department of Biotechnology, College of Science, Taif University, Taif, Saudi Arabia; ^11^ The PhD Program of Translational Medicine, College of Medical Science and Technology, Taipei Medical University, Taipei, Taiwan; ^12^ Clinical Research Center, Taipei Medical University Hospital, Taipei Medical University, Taipei, Taiwan; ^13^ TMU Research Center of Cancer Translational Medicine, Taipei Medical University, Taipei, Taiwan; ^14^ Graduate Institute of Medical Sciences, National Defense Medical Center, Taipei, Taiwan

**Keywords:** thyroid carcinoma (THCA), theranostic biomarkers, immune infiltration, epithelial-to-mesenchymal transition, t-cell exclusion

## Abstract

Papillary thyroid carcinoma (PTC) is the most prevalent endocrine malignancy with a steadily increasing global incidence in recent decades. The pathogenesis of PTC is poorly understood, and the present diagnostic protocols are deficient. Thus, identifying novel prognostic biomarkers to improve our understanding of the mechanisms of pathogenesis, diagnosis, and designing therapeutic strategies for PTC is crucial. In this study, we integrated 27 PTC transcriptomic datasets and identified overlapping differentially expressed genes (DEGs) and differentially expressed microRNAs, collectively known as thyroid tumor-enriched proteins (TTEPs), and TTEmiRs, respectively. Our integrated bioinformatics analysis revealed that TTEPs were associated with tumor stages, poor surgical outcomes, distant metastasis, and worse prognoses in PTC cohorts. In addition, TTEPs were found to be associated with tumor immune infiltrating cells and immunosuppressive phenotypes of PTC. Enrichment analysis suggested the association of TTEPs with epithelial-to-mesenchymal transition (EMT), cell-matrix remodeling, and transcriptional dysregulation, while the TTEmiRs (miR-146b-5p and miR-21-5p) were associated with the modulation of the immune response, EMT, migration, cellular proliferation, and stemness. Molecular docking simulations were performed to evaluate binding affinities between TTEPs and antrocinnamomin, antcin, and antrocin, the bioactive compounds from one of the most reputable Taiwan indigenous medicinal plants (*Antrodia camphorata*). Our results revealed that antcin exhibited higher binding efficacies toward FN1, ETV5, and NRCAM, whereas antrocin demonstrated the least. Among the targets, fibronectin (FN1) demonstrated high ligandability potential for the compounds whereas NRCAM demonstrated the least. Collectively, our results hinted at the potential of *antcin* for targeting TTEPs. In conclusion, this comprehensive bioinformatics analysis strongly suggested that TTEPs and TTEmiRs could be used as potential diagnostic biomarker signatures and be exploited as potential targets for therapeutics development.

## Introduction

Thyroid carcinoma (THCA) is a common endocrine malignancy. In recent decades, the global incidence of THCA has been on a steady increase, and for some countries, THCA is the fastest-growing malignant tumor (Chen et al., 2018). In 2020, 586,202 new cases and 43,646 deaths from THCA were reported globally, with a higher incidence in females (Sung et al., 2021). Histologically, THCA is categorized into anaplastic thyroid cancer (ATC), follicular thyroid carcinoma (FTC), and papillary thyroid carcinoma (PTC) (Luo et al., 2021). PTC and FTC are well-differentiated, accounting for more than 75% of all cases of thyroid cancers with an optimal prognosis of about 10-year disease-specific survival (Fagin and Wells, 2016). However, ATC is poorly differentiated with proliferative stem cell-like properties, resistance to present therapies (Knauf et al., 2005), and is responsible for most thyroid cancer-related deaths (Molinaro et al., 2017a). The etiology of thyroid cancer is not well understood. However, a rapid increase in PTC incidence has been primarily attributed to exposure to ionizing radiation *via* ultrasonography and diagnostic imaging modalities (Brito et al., 2013; Udelsman and Zhang, 2014). Also, accumulating evidence suggests that other factors, including hormonal exposures, obesity, metabolic syndromes, and environmental pollutants, contribute to the development of PTC-THCA (Kitahara et al., 2018).

The clinical management of PTC comprises multiple approaches, including surgery and chemo-radiotherapies (Molinaro et al., 2017b). However, accurate diagnosis before the initiation of treatment is fundamental to achieving a better prognosis for the patients. The present diagnostic protocols are suboptimal, resulting in over-diagnosing and over-treating patients with lower-risk diseases or benign thyroid nodules (Barrea, Gallo, Ruggeri, Giacinto, Sesti, Prinzi, Adinolfi, Barucca, Renzelli, Muscogiuri, et al.). At the same time, it is also essential to identify patients with high-risk and advanced stages of the disease for taking a more aggressive therapeutic approach (Durante et al., 2015). Based on these unmet clinical needs, identifying novel prognostic biomarkers represents a critical endeavor to improve our understanding of the mechanism of PTC pathogenesis, aid diagnosis, and develop therapeutic strategies.

MicroRNAs (miRNAs) are small non-coding RNAs of about 19–25 nucleotides, which regulate the translation of target mRNAs (Tanase et al., 2012). A single microRNA molecule can regulate the translation of several hundreds of genes to modulate several biological pathways (John et al., 2004). MiRNAs regulate the gene expression at the posttranscriptional level by base-pairing with complementary sequences, thereby degrading and inhibiting the translation of target mRNAs (Tran et al., 2017). Increasing evidence indicates that miRs are involved in the tumorigenesis and metastatic process of several human cancers (Farazi et al., 2013; Peng and Croce, 2016). Therefore, determining differentially expressed miRNAs between tumor and non-tumor samples has become one of the most studied areas in oncology to discover novel biomarkers for the diagnosis, prognosis, and prediction of therapeutic responses (Nikitina et al., 2012). However, because of the large number of targets that a single miRNA can regulate, large-scale transcriptomic and more comprehensive approaches are required.

The tumor microenvironment (TME), a complex system that contains cancer, immune and stroma cells, chemokines, and cytokines, has been shown to be an important player in the development of various tumors (Wu et al., 2021). The interactions of these components play an essential role in the initiation and progression of various cancers (Chen et al., 2021a). The tumor mass is infiltrated with immunosuppressive cells that vary with the host immune status and have latent prognostic value in various cancers. For instance, cancer-associated fibroblasts (CAFs), regulatory T cells (Treg), tumor-associated macrophages (TAMs), and myeloid-derived suppressor cells (MSDCs) are immunosuppressive cells that play a crucial role in suppressing the host’s anti-cancer immunological responses (Lawal et al., 2022). The aforementioned immunosuppressive cells can inhibit cytotoxic lymphocytes’ function and abundance, leading to T-cell exclusion and invasive phenotype of the cancer cells (Chen et al., 2021a). In another context, tumors are infiltrated with TAMs, and the TAMs’ immune-suppressive secretome promotes T-cell anergy and dysfunction, resulting in suboptimal T-cell differentiation, maturation, and loss of effector function (Lawal et al., 2021a). Increasing evidence indicates that genetic alterations in oncogenic genes in part can determine the infiltration of immune cells. Thus, identifying and targeting these altered oncogenes could lead to a more precise model of immunotherapy and better clinical outcomes for the patients.

With the recent advancement in sequencing technologies, many microarrays and sequencing data are deposited and available for exploring theranostic biomarkers for PTC (He et al., 2005; Giordano et al., 2006; Salvat ore et al., 2007; Vasko et al., 2007; Yu et al., 2008; Hinsch et al., 2009; Pita et al., 2009; Tomás et al., 2012; Pita et al., 2014; Reeb and Reigh-Yi, 2015; Rusinek et al., 2015; Tarabichi et al., 2015; von Roemeling et al., 2015; Handkiewicz-Junak et al., 2016; Lassalle et al., 2016; Minna et al., 2016; Romeo et al., 2018; Lee et al., 2019; Barro s-Filho et al., 2020; Colombo et al., 2020; Minna et al., 2020). However, the low sample sizes in individual studies create a considerable inter-study variability of hallmark genes with limited statistical accuracy (Chen et al., 2021b). Therefore, integrating differentially expressed genes (DEGs) from large-scale cancer transcriptomic datasets to identify intersected DEGs would provide more reliable biomarkers with unified pathogenic and prognostic relevance in PTC. This study integrated 27 transcriptomic datasets from PTC and normal samples. We identified graded levels of biomarkers associated with the immunological pathogenesis of PTC suggestive of T-cell exclusion, EMT, and cell-matrix adhesion mechanisms. We identified a synthetic compound (clopidogrel) and a peptide (ocriplasmin) as the candidate ligand inhibitors of TTEPs. Furthermore, we used molecular docking simulation of receptor-ligand interactions to explore the therapeutic potential of antrocinnamomin, antcin, and antrocin, the bioactive compounds from one of the most reputable Taiwan indigenous medicinal plants (*Antrodia camphorata*) (Geethangili and Tzeng, 2011). Our results served as a foundation for subsequent experimental validation and precision medicine approaches in managing PTC.

## Materials and methods

### Large-scale acquisition of genes and microRNA sequencing data of thyroid cancers

We queried the NCBI Gene Expression Omnibus (GEO) microarray datasets (http://www.ncbi.nlm.nih.gov/geo/) for transcriptomic data of RNA and microRNA (miRNA) profiles from PTC in comparison with normal tissue. We identified a total of 27 datasets (He et al., 2005; Giordano et al., 2006; Salvat ore et al., 2007; Vasko et al., 2007; Yu et al., 2008; Hinsch et al., 2009; Pita et al., 2009; Tomás et al., 2012; Pita et al., 2014; Reeb and Reigh-Yi, 2015; Rusinek et al., 2015; Tarabichi et al., 2015; von Roemeling et al., 2015; Handkiewicz-Junak et al., 2016; Lassalle et al., 2016; Minna et al., 2016; Romeo et al., 2018; Lee et al., 2019; Barro s-Filho et al., 2020) (Colombo et al., 2020; Minna et al., 2020) comprising 22 RNA and five miRNA sequencing datasets (Table 1). The dataset containing primary or metastatic cancer tissues (tumor samples) and normal human samples (normal counterparts) was included. Controls were normal healthy cohorts with no cancer. No ethics committee approval or patient consent was required for the present study. The datasets were analyzed for differentially expressed genes (DEGs) and differentially expressed microRNAs (DEmiRNAs) between the cancer and normal tissues using the LIMMA package of R based on the selection cutoff points of logFC: >1.5 and *p*-value adjusted Benjamini–Hochberg correction and false discovery rate (FDR) < 0.05. The DEGs that met these criteria were sorted to identify the intersected DEGs from all datasets using the Excel lookup and data sorting. The online tool Multiple List Comparator (https://www.molbiotools.com/listcompare.html) was used to visualize the intersected DEGs and generate a Venn diagram for the visualization of the overlapping DEmiRNAs. Furthermore, the DEGs clustering from each dataset were generated using the ImageGP visualization tools (http://www.ehbio.com/ImageGP/). In addition, we also explored The Cancer Genome Atlas (TCGA) dataset of THCA, which comprises 507 cohorts with papillary thyroid carcinoma (PTC).

**TABLE 1:** Characteristics of the datasets used for the large-scale acquisition of differentially expressed genes (DEGs) andmicroRNAs (miRs) in thyroid cancers.

	GSE accession	GPL	THCA	Normal	Release date	References
1	GSE3467	GPL570 (HG-U133_Plus_2) Affymetrix human genome U133 plus 2.0 array	9	7	Dec 19, 2005	He et al. (2005)
2	GSE3678	GPL570 (HG-U133_Plus_2) Affymetrix human genome U133 plus 2.0 array	7	7	Jun 30, 2006	
3	GSE33630	GPL570 (HG-U133_Plus_2) Affymetrix human genome U133 plus 2.0 array	60	45	Nov 09, 2012	Tomás et al. (2012)
4	GSE58545	GPL96 (HG-U133A) Affymetrix human genome U133A array	27	18	Dec 31, 2015	Rusinek et al. (2015)
5	GSE65144	GPL570 (HG-U133_Plus_2) Affymetrix human genome U133 plus 2.0 array	12	13	Jan 22, 2015	von Roemeling et al. (2015)
6	GSE29265	GPL570 (HG-U133_Plus_2) Affymetrix human genome U133 plus 2.0 array	29	20	Jun 01, 2012	
7	GSE53072	GPL6244	3	3	Dec 07, 2013	Pita et al. (2014)
8	GSE35570	GPL570 (HG-U133_Plus_2) Affymetrix human genome U133 plus 2.0 array	65	51	Dec 31, 2015	Handkiewicz-Junak et al. (2016)
9	GSE60542	GPL570 (HG-U133_Plus_2) Affymetrix human genome U133 plus 2.0 array	58	32	Sep 01, 2015	Tarabichi et al. (2015)
10	GSE29315	GPL8300 (HG_U95Av2) Affymetrix human genome U95 version 2 array	31	25	Jun 01, 2012	
11	GSE6004	GPL570 (HG-U133_Plus_2) Affymetrix human genome U133 plus 2.0 array	14	4	Oct 11, 2006	Vasko et al. (2007)
12	GSE27155	GPL96 (HG-U133A) Affymetrix human genome U133A array	95	4	Feb 09, 2011	Giordano et al. (2006)
13	GSE9115	GPL5917 (human 12K cDNA clones, print AgB3)	15	5	Sep 21, 2007	Salvat ore et al. (2007)
14	GSE82208	GPL570 (HG-U133_Plus_2) Affymetrix human genome U133 plus 2.0 array	27	25	Jun 03, 2017	
15	GSE129562	GPL10558 (Illumina Human) HT-12 V4.0 expression beadchip	8	8	Nov 12, 2019	Lee et al. (2019)
16	GSE85457	GPL570 (HG-U133_Plus_2) Affymetrix human genome U133 plus 2.0 array	4	3	Nov 30, 2016	
17	GSE5364	GPL96 (HG-U133A) Affymetrix human genome U133A array	35	16	Jul 23, 2008	Yu et al. (2008)
18	GSE53157	GPL570 (HG-U133_Plus_2) Affymetrix human genome U133 plus 2.0 array	24	3	Dec 10, 2013	Pita et al. (2009)
19	GSE58546	GPL570 (HG-U133_Plus_2) Affymetrix human genome U133 plus 2.0 array	10	10	Dec 31, 2015	Rusinek et al. (2015)
20	GSE53050	GPL570 (HG-U133_Plus_2) Affymetrix human genome U133 plus 2.0 array	3	3	Dec 31, 2015	Reeb and Reigh-Yi (2015)
21	GSE50901	GPL13607 (Agilent-028004 surePrint G3 human GE 8 × 60 K microarray]	61	4	Nov 01, 2014	Barro s-Filho et al. (2020)
22	GSE15045	GPL2986 (ABI human genome survey microarray version 2)	8	4	Feb 28, 2009	Hinsch et al. (2009)
23	GSE73182	GPL20194: Agilent-035758 human miRBASE 16.0 plus 031181	16	5	Nov 01, 2016	Minna et al. (2016)
24	GSE97070	GPL18402: Agilent-046064 human_miRNA_V19.0	17	3	Mar 01, 2018	Romeo et al. (2018)
25	GSE40807	GPL:Agilent-019118 human miRNA microarray 2.0 G4470B	40	40	Dec 15, 2014	Lassalle et al. (2016)
26	GSE151180	GPL21575:Agilent-070156 human_miRNA_V21.0_microarray 046064	16	11	May 17, 2021	Colombo et al. (2020)
27	GSE104006	GPL20194: Agilent-035758 human miRBASE 16.0 plus 031181	29	5	Sep 30, 2020	Minna et al. (2020)

### Differential expressions of thyroid tumor-enriched proteins in The Cancer Genome Atlas-thyroid carcinoma cohorts

We analyzed differential expression levels of the TTEPs between tumor and adjacent normal tissues of The Cancer Genome Atlas (TCGA) cohorts of the PTC dataset using the Tumor Immune Estimation Resource (TIMER, version 2.0) (Li et al., 2017). Furthermore, the TTEP sets were analyzed for gene set variation scores between TCGA thyroid tumor and adjacent normal tissue, and also between four types of thyroid tumor stages, namely, the well-differentiated (low grade, stage I), moderately differentiated (intermediate grade, stage II), poorly differentiated (high grade, stage III), and undifferentiated (high grade, stage IV). Differential expression was considered statistically significant at *p* < 0.05, <0.01, and <0.001.

### Differential methylation of thyroid tumor-enriched proteins in The Cancer Genome Atlas-thyroid carcinoma cohorts

We explored the methylation module of the GSCALite server (Liu et al., 2018) to analyze the TTEPs’ methylation differences between the thyroid tumor and paired normal tissues in TCGA database. The differential methylation between tumor and normal samples was compared using Student’s *t*-test with *p*-value adjusted by FDR ≤ 0.05. In addition, we analyzed the effect of methylation on the expression levels of TTEPs by assessing the mRNA expression correlation with methylation levels using Pearson’s correlation coefficient and the t-distribution with an FDR-adjusted *p*-value.

### Analysis of thyroid tumor-enriched proteins’ genetic alterations, and prognostic and clinical attributes in thyroid carcinoma

We used the cBioPortal tool (http://www.cbioportal.org/) (Cerami et al., 2012) to analyze the genomic alterations, survival analysis, and clinical attributes, and perform group comparisons of TTEPs in 508 patients (516 samples) of TCGA-PTC cohorts. Based on the genetic alteration profile of the cohorts, we split the cohorts into two groups, the TTEP altered and non-altered cohorts, and used the Kaplan–Meier survival plot to assess the overall survival (OS) and disease-free survival (DFS). We also compared these groups for several clinical attributes, including metastasis, response to surgery, and new neoplasm event after initial therapy using the chi-squared statistical test at *p*-value < 0.05. The analyzed results were considered significant when *p* < 0.05.

### Analysis of thyroid tumor-enriched proteins’ regulation of the abundance of immune and immuno-suppressive cell infiltrations in thyroid carcinoma

The regulation of the abundance of immune cell infiltration of PTC by the TTEPs was evaluated by analyzing the correlation between the mRNA expression levels of the TTEPs and infiltration of immunosuppressive cells that are known to promote T-cell exclusion; vis myeloid-derived suppressor cell (MDSCs), cancer-associated fibroblast (CAF), tumor-associated macrophages (M2-TAM), and regulatory T cells (Treg) using the Tumor IMmune Estimation Resource (TIMER2.0) (http://timer.cistrome.org/) server (Li et al., 2020). Furthermore, the GSCALite server (Liu et al., 2018) was used to analyze the correlation between the expression levels of the TTEPs and the tumor infiltrations of cytotoxic lymphocytes (B cells, CD4 naïve, CD4 T, CD8 naïve, CD8 T, MAIT cells, NK cells, and γδ T cells), T helper cells (Th1, Th2, Th17, and Tfh), neutrophils, dendritic cells, macrophages, and monocytes. The correlation analysis was conducted using the purity-corrected partial Spearman’s Rho value and statistical significance (*p* < 0.05) while the correlation data were visualized using the heatmap constructed with GraphPad Prism software (version 8.0.0 for Windows).

### Interaction and functional enrichment network analysis of the thyroid tumor-enriched genes

We used the IntAct molecular interaction database (https://www.ebi.ac.uk/intact/home) to analyze the direct protein–protein interaction of the TTEPs while the protein interaction network analysis (PINA) platform was used to analyze the thyroid cancer-specific protein–protein interaction network (https://omics.bjcancer.org/pina/home.action). The thyroid-specific protein interaction network was constructed based on the cutoff point of the tumor type-specific score of two and spearman correlation of 0.5. In addition, the GENEMANIA (https://genemania.org/), an online gene interaction platform (Mostafavi et al., 2008), was used to analyze the gene–gene interaction network of the TTEPs. The Enrichr, an online gene set enrichment analysis (GSEA) server (Chen et al., 2013; Kuleshov et al., 2016) and the search tool for retrieval of interacting genes/proteins (STRING) server (http://string-db.org/, v10.5) were used to analyze functional enrichment profiles of the TTEPs. The official gene symbols of the DEGs were directly uploaded into the Enrichr server and analyzed for the Kyoto Encyclopedia of Genes and Genomes (KEGG) and Gene Ontology (GO) terms under the default enrichment cutoff value of *p* < 0.05. The Entrez gene symbols of the DEGs were uploaded to the multiple protein modules of the STRING server and analyzed for known and predicted PPI interactions in *Homo sapiens* under the high confidence (0.70) search and at a significant level of *p* < 0.05. The significantly (*p* < 0.05) enriched terms of KEGG and GO)biological processes and molecular functions from the two servers were integrated and visualized using the ImageGP visualization tools.

### Thyroid tumor-enriched protein-mediated drug sensitivity analysis

Gene expression-induced drug sensitivity against different cancer cell lines was evaluated using the GSCALite (http://bioinfo.life.hust.edu.cn/web/GSCALite/) server (Liu et al., 2018). The Spearman correlation was used to correlate the messenger (m) RNA expression levels of the TTEPs and the 50% inhibitory concentration (IC_50_) of the small molecules against various cells in the Cancer Therapeutics Response Portal (CTRP) and Genomics of Drug Sensitivity (GDSC) databases.

### Interactions, functional, and disease enrichment network analysis of thyroid tumor-enriched microRNAs

We used the miR-TV database (http://mirtv.ibms.sinica.edu.tw/index.php) to evaluate the differential expression levels of mature miRNA between TCGA thyroid cancer and normal tissue from TCGA version 18.0. We also explored the miRTarBase (https://mirtarbase.cuhk.edu.cn/), an experimentally validated microRNA-target interaction database to identify the gene targets of the thyroid tumor-enriched microRNAs (TTEMiRs). While the disease and functional enrichment of the TTEMiRs were analyzed using the miRNA Enrichment Analysis and Annotation (miEAA) tool (https://ccb-compute2.cs.uni-saarland.de/mieaa2/), analysis was conducted using FDR (Benjamini–Hochberg), a *p*-value adjustment of 0.05 and a minimum required hit of two miRNAs. The gene ontology (GO) enrichment of the miRNAs was analyzed using the miRDB (http://mirdb.org/mirdb/index.html), an online database for miRNA target prediction and functional annotations (Chen and Wang, 2019). Enrichment analysis was conducted at FDR *p* < 0.05.

### Molecular docking studies of receptor-ligand interactions

The predocking and docking analysis was conducted *via* the AutoDock Vina (version 0.8, Scripps Research Institute, La Jolla, CA, United States). The three-dimensional (3D) structure of the proteins FN1 (PDB:3MQL), ETV5 (PDB:5ILV), and NRCAM (PDB:1UEN) was downloaded from the protein data bank (PDB). The mol2 files of the 3D structure of compounds were obtained using the Avogadro molecular builder and visualization tool version 1. XX (Hanwell et al., 2012) and converted to PDB format using the PyMOL Molecular Graphics System, version 1.2r3pre. The PDB files of the crystal structures of the targets were transformed to pdbqt format using AutoDock Vina (version 0.8, Scripps Research Institute, La Jolla, CA, United States) (Trott and Olson, 2010). Ligand and receptor preparations for docking were conducted as described previously (Lawal et al., 2021b; Lawal et al., 2021c; Alorabi et al., 2022; Onikanni et al., 2022). In contrast, molecular docking was performed using AutoDock Vina (version 0.8) (Trott and Olson, 2010) with all parameters set to default values, and all bonds in the ligand rotated freely while considering the receptor to be rigid. A spacing of 1.0 Å and a grid box of 40 Å × 40 Å × 40 Å in the X, Y, and Z dimensions were used (Wu et al., 2022). All docking was performed at energy range = 4 and exhaustiveness of 8. The docked complexes were generated in PDF format using the pyMOL software. The complexes were analyzed for interaction affinities between the ligand and the receptors and were expressed as binding energy values in Kcal/mol. The interaction distance of the conventional hydrogen bonding between the amino acid residues of the receptors and the ligand atoms was also analyzed and expressed in Angstrom (Å). The 2D conformation of the receptor-ligand complex was visualized using the Discovery studio visualizer version 19.1.0.18287 (BIOVIA, San Diego, CA, United States) (Visualizer, 2020).

## Results

### Large-scale transcriptomic data analysis identified graded levels of biomarker signatures of thyroid cancer pathogenesis

**FIGURE 1:**
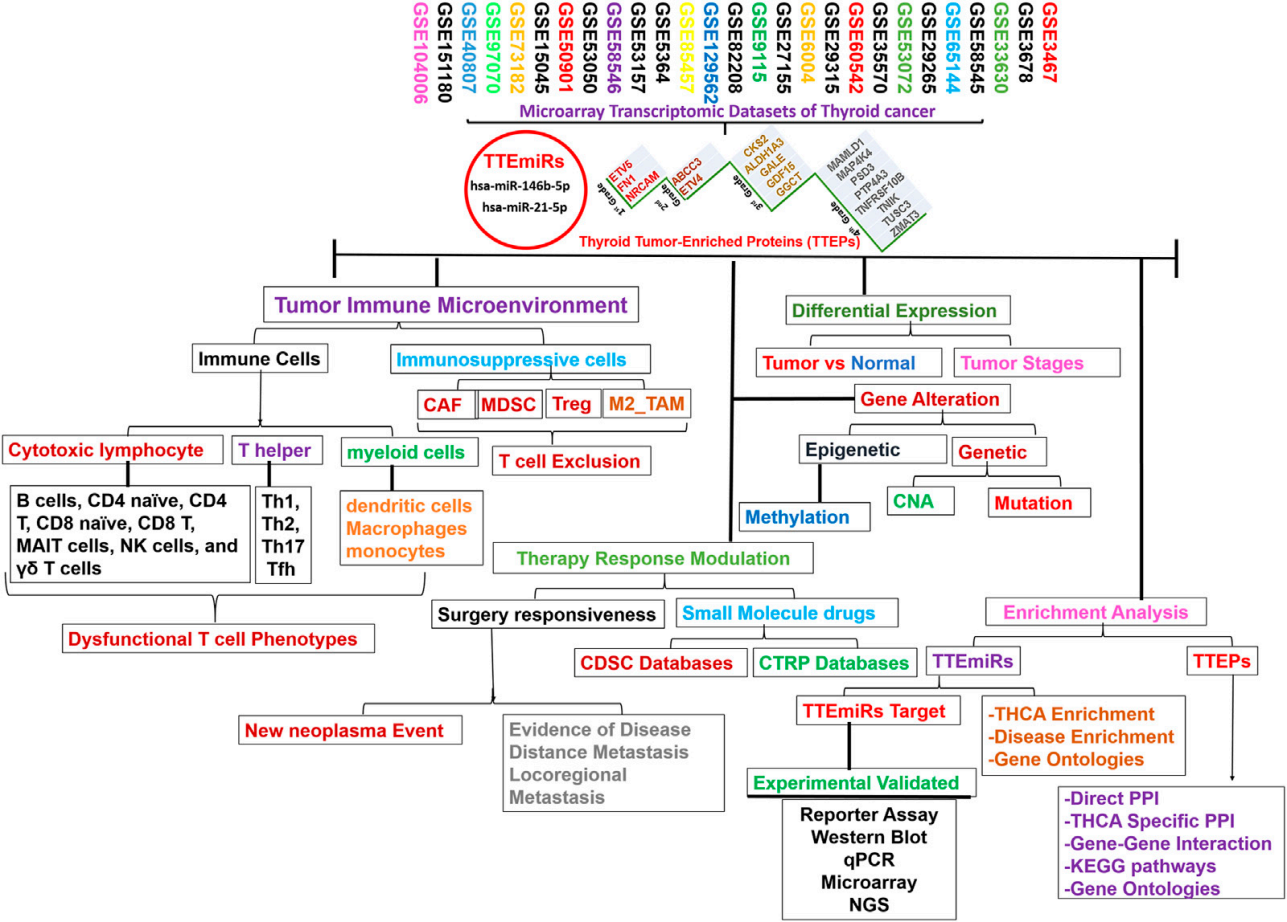
Schematic flow chart of the study design.

**FIGURE 2:**
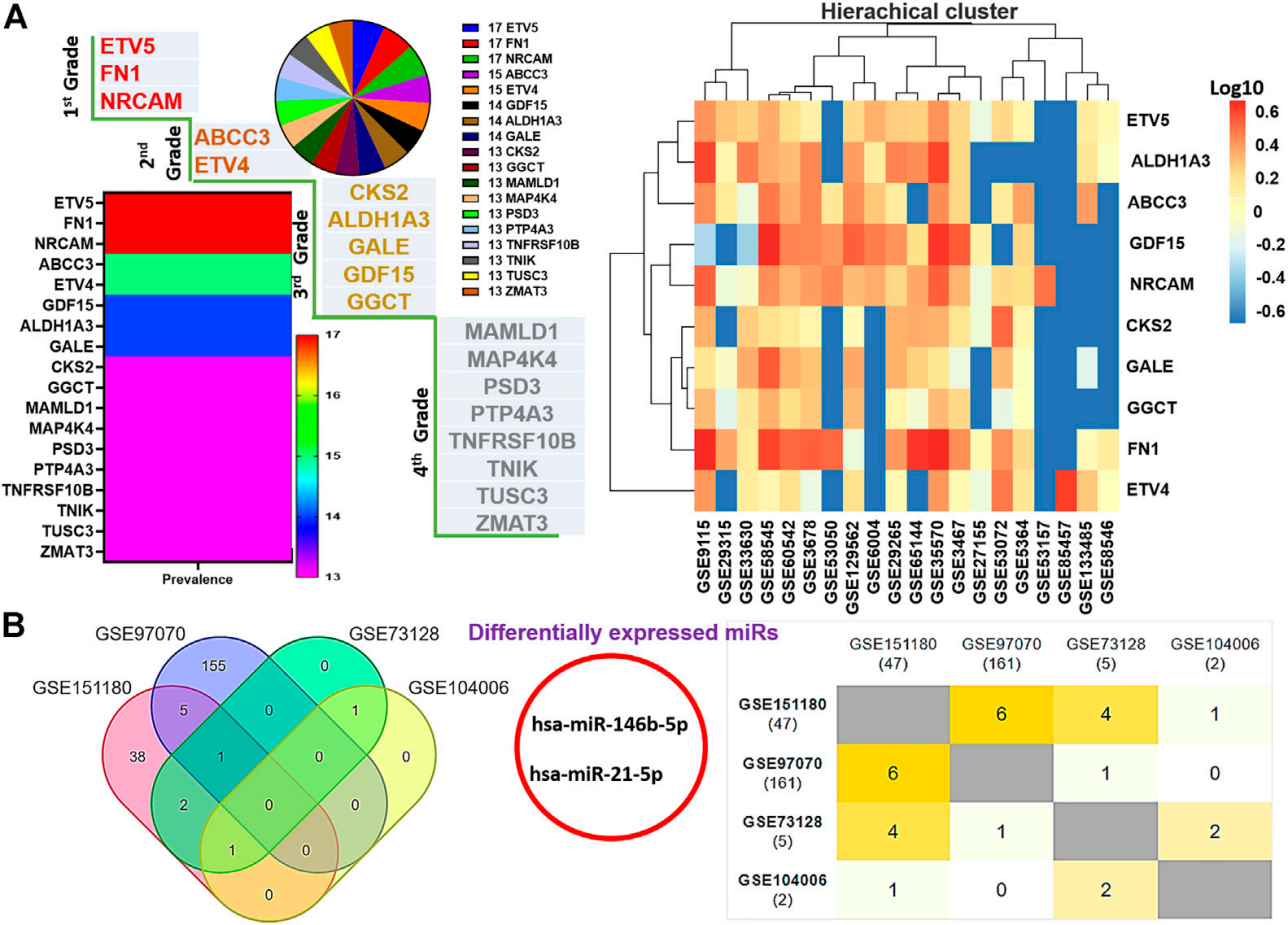
Large-scale transcriptomic data analysis identified graded levels of biomarker signatures of thyroid cancer pathogenesis. **(A)** Multiplot and hierarchical clustering of differentially expressed genes (DEGs) identified from the large-scale transcriptomic datasets. **(B)** Venn diagram and Jaccard plot of the differentially expressed microRNAs identified from the integrations of DEmiRs in THCA datasets.

**FIGURE 3:**
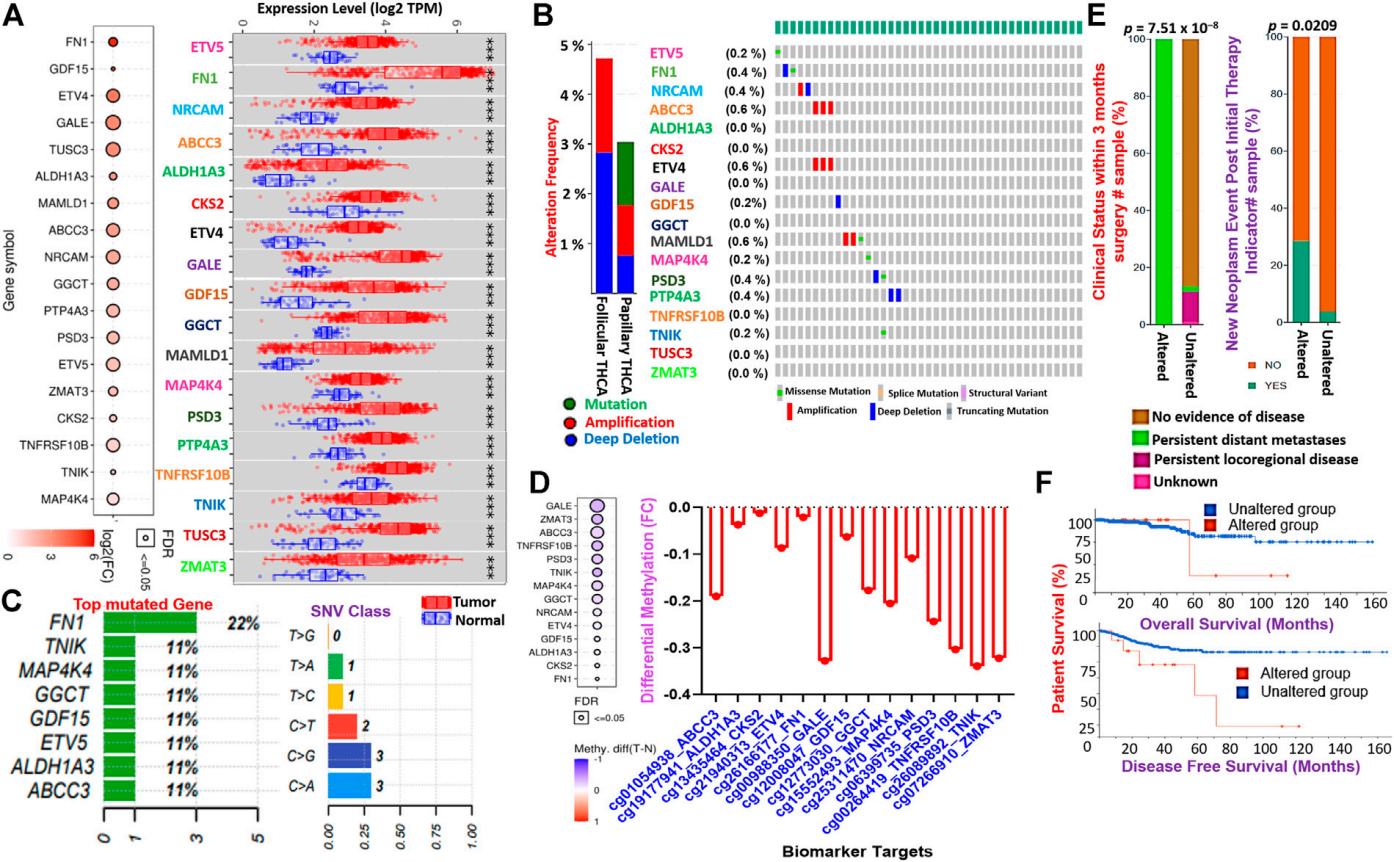
TTEPs are associated with poor surgical outcome, distant metastasis, and poor prognosis in THCA cohorts. **(A)** Bubble and boxplots of the mRNA expression levels of the TTEPs between TCGA thyroid tumor and adjacent normal tissues. **(B)** Bar and waterfall of the genetic alteration profile and **(C)** mutation plots of the TTEPs in TCGA pan-cancer of THCA cohorts. **(D)** Bubble and bar plots of the methylation status of the TTEPs in TCGA-THCA cohorts. **(E)** Bars plot of the clinical attribute of TCGA-THCA cohorts with genetically altered TTEPs. **(F)** Kaplan–Meier plots of the overall and disease-free survival differences between TCGA-THCA cohorts with genetically altered and wild-type TTEPs.

**FIGURE 4:**
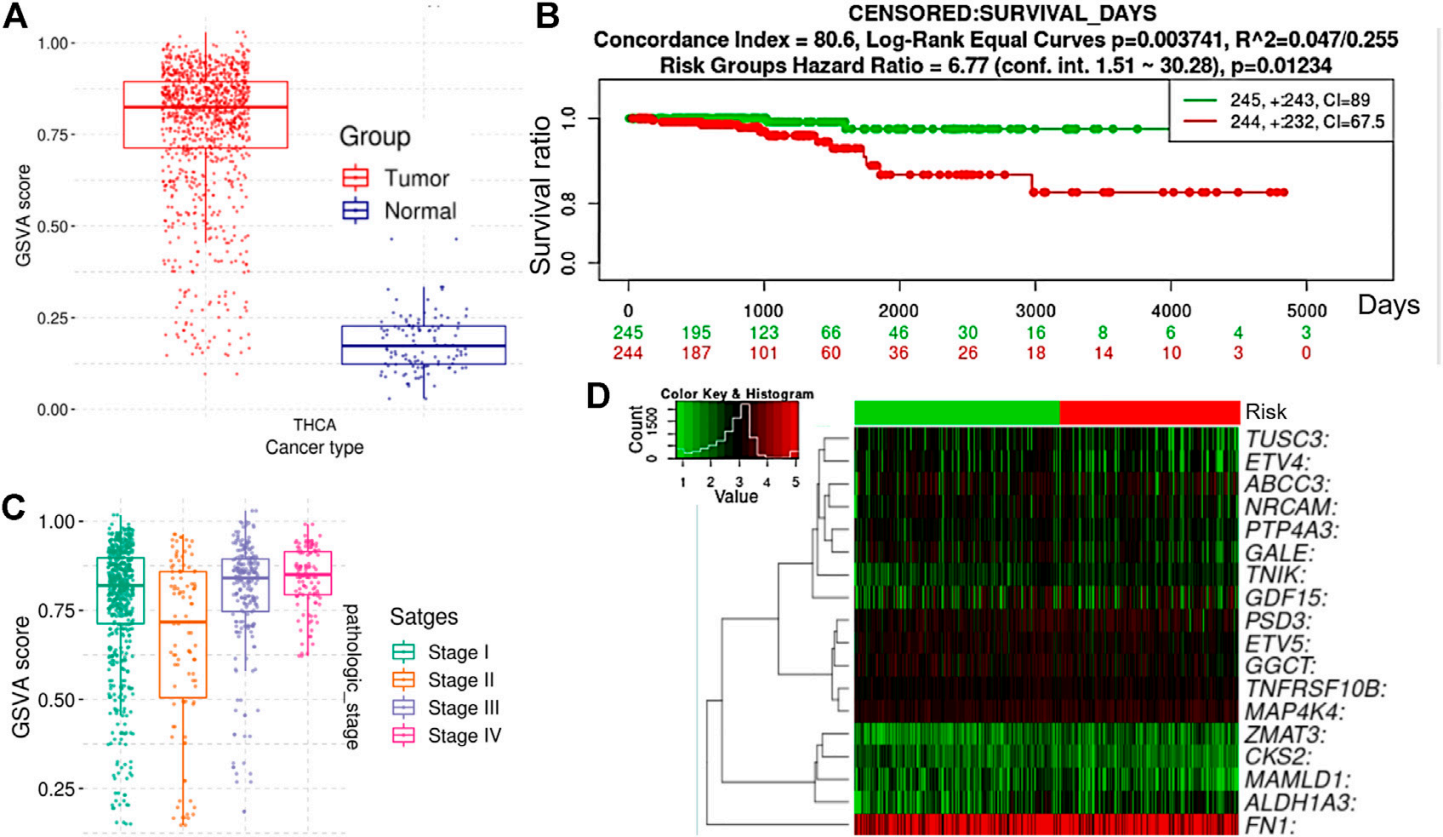
Gene enrichment analyses of the TTEPs from TCGA-PTC cohorts. **(A)** Bar plot of the gene set variation analysis (GSVA) score of TTEPs between TCGA-PTC cohorts and adjacent normal tissue. The GSVA is a GSE method that estimates the variation of pathway activity over a sample population in an unsupervised manner. Compared to the normal tissue, the TTEPs achieved a very high GSVA score, which shows a negative GSVA score in PTC. **(B)** Bar plots of the differential GSVA score between tumor stages. **(C)** Kaplan–Meier plot, and **(D)** risk plot of TCGA-PTC cohorts with high and low-expression profiles of TTEPs. The PTC cohorts with high expression levels of TTEPs exhibited high hazard risk and shorter survival duration when compared with cohorts with low expression levels of the TTEPs.

**FIGURE 5:**
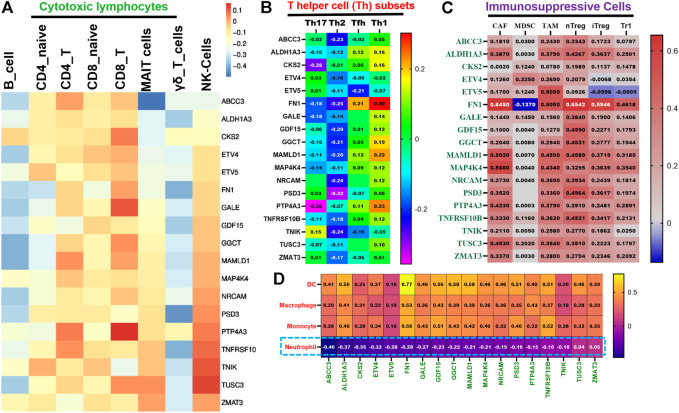
TTEPs demonstrated a significant association with the T-cell exclusion mechanism of immune-invasive phenotypes in THCA. Heatmap plots of the mRNA association with tumor in infiltrations of cytotoxic lymphocytes **(A)**, T-helper cells **(B)**, immunosuppressive cells **(C)**, myeloid cells, and neutrophils **(D)** in TCGA-THCA cohorts.

**FIGURE 6:**
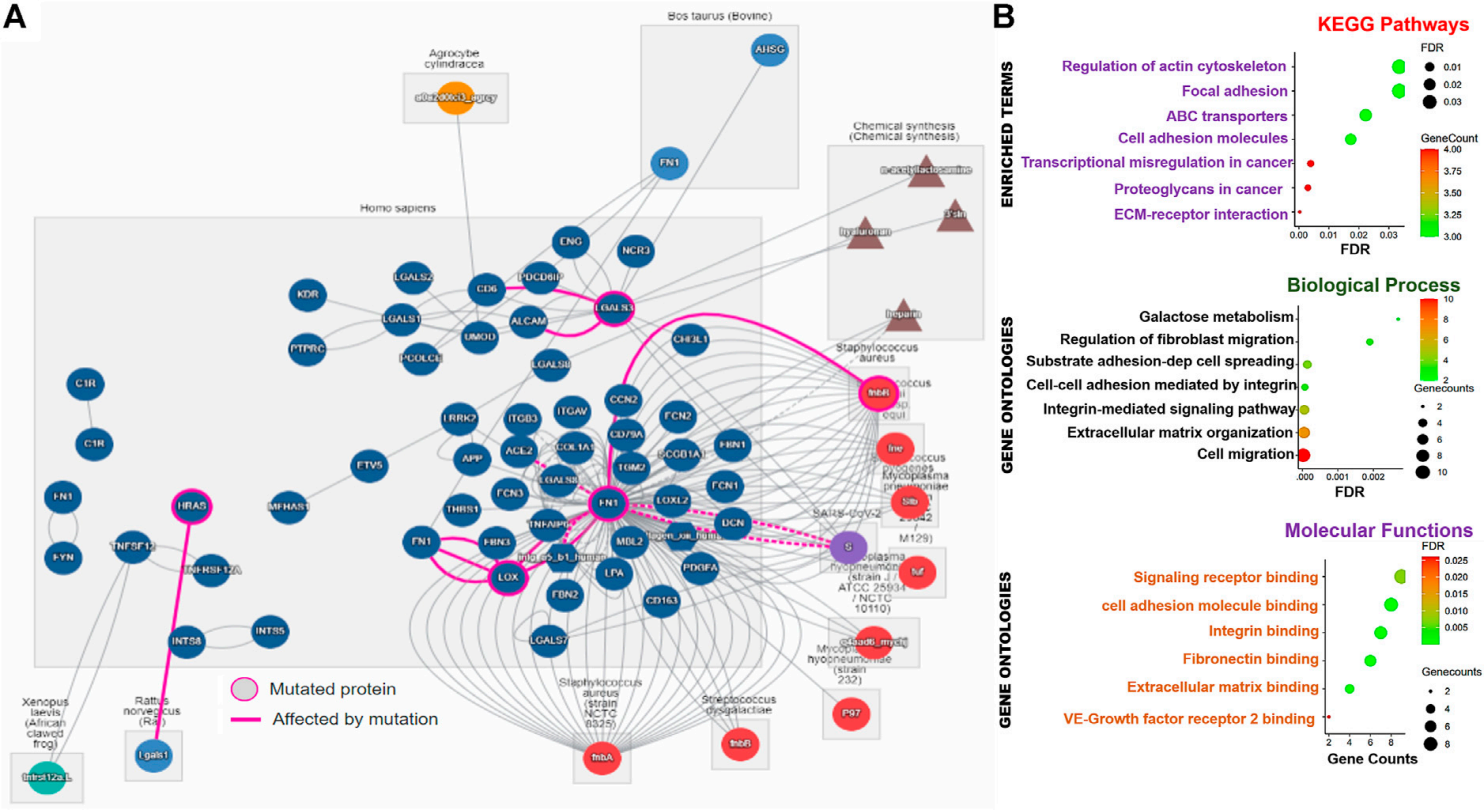
Thyroid tumor-enriched proteins (TTEPs) induced significant pathological interactions *via* cell-matrix adhesion and transcriptional dysregulation. **(A)** Network plots of direct protein–protein interactions of TTEPs. The direct protein–protein interaction was queried based on the human interaction of the proteins under the influence of mutation or chemical treatment. **(B)** Enrichment network plots of KEGG pathways, GO molecular functions, and biological processes of the TTEPs.

**FIGURE 7:**
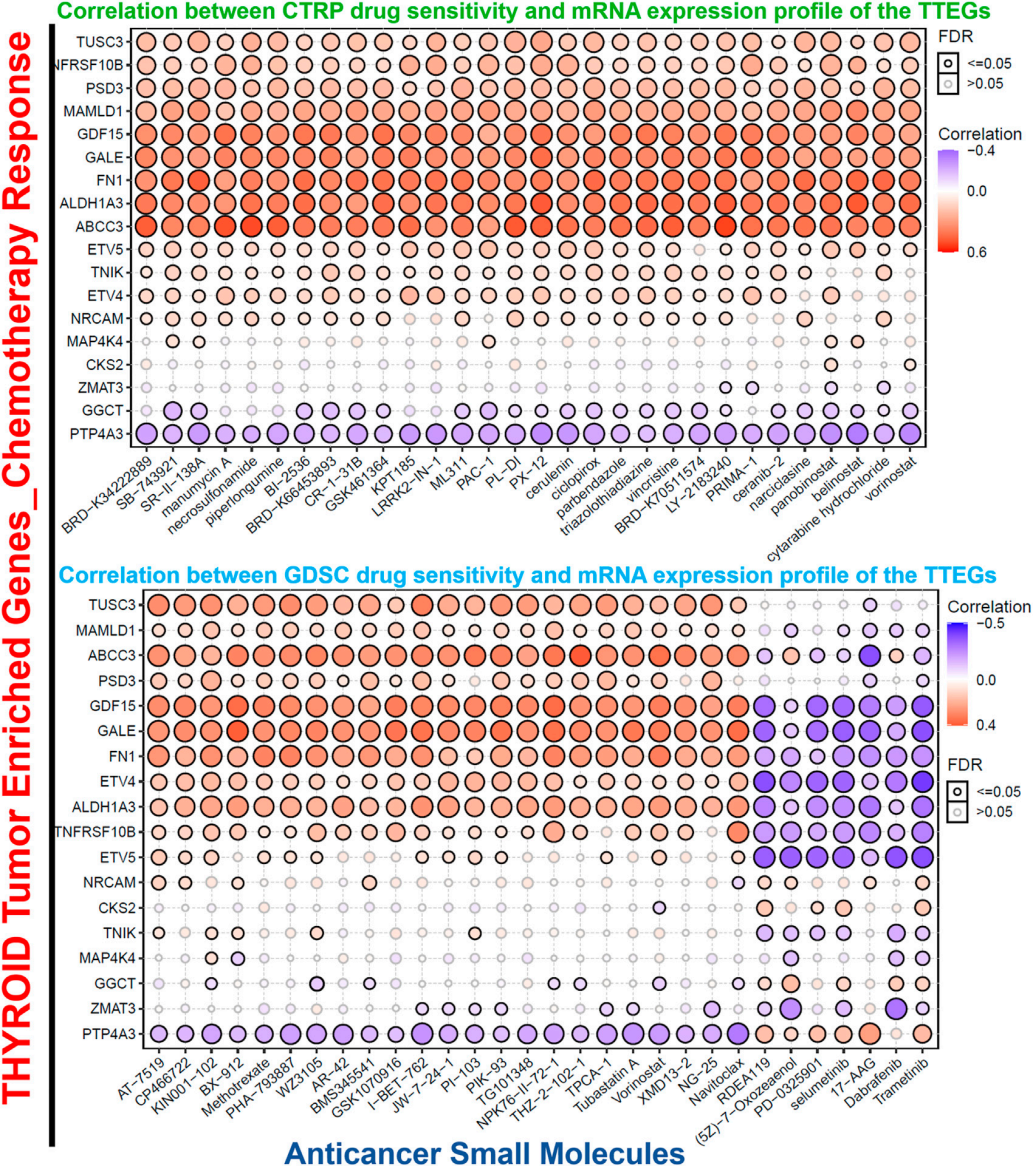
Bubble plots of TTEPs’ association with the sensitivity of various GDSC and CTRP anti-cancer drugs.

The flowchart of the study is shown in Figure 1. We integrated DEGs from large-scale datasets of thyroid cancer to identify graded levels of biomarker signatures of thyroid cancer pathogenesis (Figure 2A). Based on the number of datasets at which each DEG was identified and the prognostic role of the DEGs, we categorized the DEGs as grade 1 (ETV5, FN1, and NRCAM), grade 2 (ABCC3 and ETV4), grade 3 (ALDH1A3, CKS2, GALE, GDF15, and GGCT), and grade 4 (MAMLD1, MAP4K4, PSD3, PTP4A3, TNFRSF10B, TNIK, TUSC3, and ZMAT3) levels of biomarker signatures of thyroid cancer pathogenesis. Collectively, these DEGs are termed tumor thyroid-enriched genes (TTEPs) and were used for the subsequent analysis of the mechanisms of thyroid carcinogenesis. Furthermore, the integration of differentially expressed microRNAs (DEmiRs) from four other GEO datasets (GSE97070, GSE151180, GSE73128, and GSE104006) of miRNAs in thyroid cancers (Figure 2B) identified only two intersected miRNAs, miR-21-5p and miR-146b-5p, collectively termed thyroid tumor-enriched miRs (TTEmiRs). Altogether, our large-scale transcriptomic data analysis identified graded levels of biomarker signatures of thyroid cancer pathogenesis, and our further analysis (Figures 3–7) suggested that these signatures modulate specified genetic signaling and are thyroid cancer-specific, and hence, could be useful in the clinical diagnosis of thyroid cancer.

### Thyroid tumor-enriched proteins are associated with tumor stages, poor surgical outcome, distance metastasis, and worse prognosis

To validate our findings from the large-scale transcriptomic data analysis, we used TCGA cohorts to evaluate the differential expression levels of the TTEPs between the cohorts’ tumor tissue and adjacent normal tissue. Notably, we found that all members of the TTEPs were significantly (*p* < 0.001) overexpressed in the thyroid tumor tissue compared with the adjacent normal tissues (Figure 3A). In addition, the TTEPs exhibited various types of genetic alterations (mutation, amplification, and deep deletion) in thyroid cancer (Figures 3B,C), with the grade 1 signature having the highest occurrence of genetic mutations (Figure 3C). Consistently, the TTEPs were found to be significantly hypomethylated in THCA tumors compared with the normal tissues (Figure 3D). Interestingly, we found that thyroid cancer patients with genetic alterations of TTEPs exhibited persistent distant metastases within 3 months of surgery (Figure 3E). In addition, this group of thyroid cancer patients demonstrated significantly (*p* < 0.0209) higher levels of new neoplasm events after initial therapy (Figure 3E) and worse prognosis as measured by the overall survival and disease-free survival (Figure 3F). TTEPs exhibited high enrichment and GSVA (gene set variation analysis) scores in PTC compared with adjacent non-tumor tissues (Figure 4A). In addition, the expression levels of the TTEPs are significantly associated with tumor stages (Figure 4B), high risk, and shorter survival duration of the cohorts (Figures 4C,D). The GSVA of the TTEPs revealed a strict activation of EMT among 10 other pathways (Supplementary Figure S1) and immunosuppressive cells of invasive tumor phenotypes (Supplementary Figure S2) in PTC. Collectively, our results demonstrated that the TTEPs’ expression increased with disease stages (except for stage 2), and is associated with a poor surgical outcome, increased incidence of distance metastases, and poor prognosis in clinical PTC cohorts.

### Thyroid tumor-enriched proteins demonstrated a unified association with the T-cell exclusion mechanism of immune-invasive phenotypes of thyroid carcinoma

Our analysis revealed that all members of TTEPs demonstrated a cooperative association with the infiltration levels of different categories of immune and immunosuppressive cells in THCA (Figures 5A–D). We found that the mRNA expression levels of the TTEP showed no significant (*p* > 0.05) positive association with the infiltration levels of cytotoxic lymphocyte (Figures 5A,B) cells, CD4 naïve, CD4 T, CD8 naïve, CD8 T, MAIT cells, NK cells, and γδ T cells, and significant negative association (*p* < 0.05) with the infiltration of T-helper cells (Figure 5B) and neutrophils (Figure 5D). However, the mRNA expression levels of the TTEPs show a significant and positive (*p* < 0.05) association with the infiltration levels of immunosuppressive cells (CAF, MDSC, TAM, nTreg, iTreg, and Tr1) (Figure 5C), dendritic cells, macrophages, and monocytes (Figure 5D). These immunosuppressive phenotypes are markers of T-cell exclusion. Hence, our results strongly suggested that TTEPs exhibited a unified association with the T-cell exclusion mechanism of immune-invasive phenotypes in PTC.

### Thyroid tumor-enriched proteins are significantly associated with cell-matrix adhesion mechanisms and transcriptional dysregulation in thyroid carcinoma

We conducted a series of functional and interaction analyses to gain further insight into the potential pathological mechanisms of the TTEP**.** Notably, the direct protein–protein interaction analysis revealed a total of 74 interactors and 181 direct interactions with the targets, of which FN1 occupied a total of 126 interactions. In addition, FN1 serves as the major mutated protein of the network, affecting the biological roles of LOX, ACE2, DCN, ITGAV, and FNbB (Figure 6A). Thyroid cancer-specific PPI of the targets identified 121 unique disease-related interactions with the targets (Supplementary Figure S3). Specifically, we found that the interactions of the DEGs with SPTBN1, TUBB2A, ANK2, CEP350, PCLO, CDK12, MRPS16, TUBB2B, and IL20RA, were significantly associated with poor prognosis of the PTC cohorts (Supplementary Figure S3). Furthermore, we identified 38 gene–gene interacting networks significantly involved in membrane/cell ruffling, leading-edge membrane, cell division site, and lyse activity (Supplementary Figure S3). Similarly, our gene set enrichment analysis (GSEA) demonstrated that thyroid tumor-enriched proteins were significantly enriched in KEGG pathways, including the ABC transporters, transcriptional misregulation in cancer, extracellular matrix (ECM) receptor interaction, proteoglycans in cancer, cell adhesion molecules, and focal adhesion and regulation of the actin cytoskeleton. The enriched gene ontologies of the TTEP included cell migration, extracellular matrix organization, integrin-mediated signaling pathway, cell–cell adhesion mediated by integrin substrate adhesion-dependent cell spreading, regulation of fibroblast migration, and galactose metabolism (Figure 6B). Collectively, our results suggested that the thyroid tumor-enriched proteins (TTEP) induced significant pathological interactions associated with tumor progression and worse prognosis *via* the cell-matrix adhesion mechanism and transcriptional dysregulation.

### Thyroid tumor-enriched proteins mediate antitumor drug resistance and unique association with cancer cell response to MEK-inhibitors

Scientific evidence has indicated that the expression of several oncogenic genes is associated with drug resistance in various cancers (Gillet et al., 2011). On this premise, we analyzed the relationship between the levels of TTEP expression and the response of cancer cell lines to several chemotherapies. The data obtained revealed that with the exclusion of PTP4A3 and GGCT, and the levels of TTEP expressions were significantly (*p* < 0.05) associated with the resistance of cancer cell lines to several classes of small molecules of anti-cancer functions. On the contrary, we observed a distinct association with MEK inhibitors; these gene signatures induced increased sensitivity of the cancer cell lines to numerous inhibitors of MEK, including trametinib, selumetinib, 17-AAG, RDEA119, PD-0325901, (5Z)-7-oxozeaenol, and a mutant-BRAF kinase inhibitor, dabrafenib (Figure 7).

### Identification of miR-146b-5p and miR-21-5p as thyroid carcinoma-specific oncogenic microRNA

**FIGURE 8:**
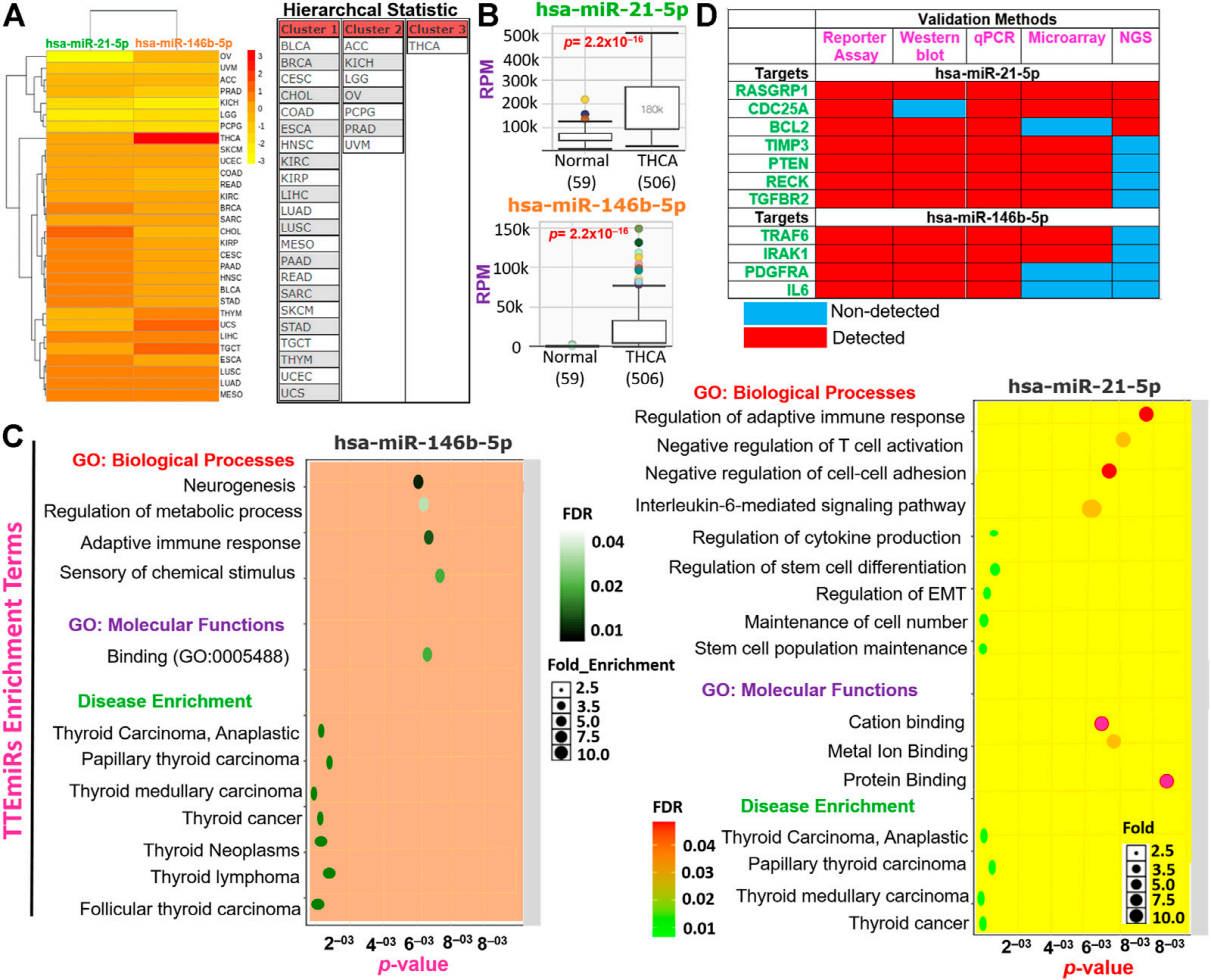
Thyroid tumor-enriched microRNA (TTEmiRs) are THCA-specific oncogenic microRNA (miRs) that modulate the immune response, EMT, cell–cell adhesion to promote cell growth, and stem cell population. **(A)** Heatmap plot and hierarchal clustering of the TTEmiRs in TCGA cancer cohorts. **(B)** Bar plots showing the differential expression levels of the TTEmiRs between TCGA-THCA tumor and adjacent normal tissue and **(C)** enrichment plots of the TTEmiRs. **(D)** Plots of the TTEmiRs target genes based on experimentally validated protocols **(D)**.

We evaluated and compared the hierarchal clustering and differential expression profiles of miR-146b-5p and miR-21-5p between TCGA cohorts of THCA and healthy tissues. We found that, of the 33 TCGA cancer cohorts, miR-146b-5p and miR-21-5p formed three clusters (Figure 8A). Notably, both miRs were found significantly (*p* = 2.2 × 10^–16^) overexpressed in THCA compared to the adjacent normal tissues (Figure 8B). However, our dataset analysis of differential miRNA expression between tumor histology revealed no significant differential expression of these miRs among the different THCA histology. The subsequent enrichment analysis revealed that miR-146b-5p and miR-21-5p were specifically associated with the regulation of immune response, EMT, cell–cell adhesion, cell growth, stem cell population maintenance, and the progression of several thyroid-associated diseases (Figure 8C). More importantly, through exploration of experimental evidence from Western blot, reporter assay, qPCR, NGS, and microarray, RASGRP1, CDC25A, BCL2, TIMP3, PTEN, RECK, TGFBR2, TRAF6, IRAK1, PDGFRA, and IL6 were identified as the major target genes of miR-146b-5p and miR-21-5p in THCA (Figure 8D). Consistently, we found that the aforementioned genes were significantly downregulated in THCA compared with adjacent normal tissues. Our analyses strongly suggested that miR-146b-5p and miR-21-5p (which we termed TTEmiRs) are THCA-specific oncogenic miRs through immune modulation and EMT induction to promote cellular adhesion/growth, and stem cell population maintenance in THCA.

### Potential drug target exploration and molecular docking of target-ligand simulation

**FIGURE 9:**
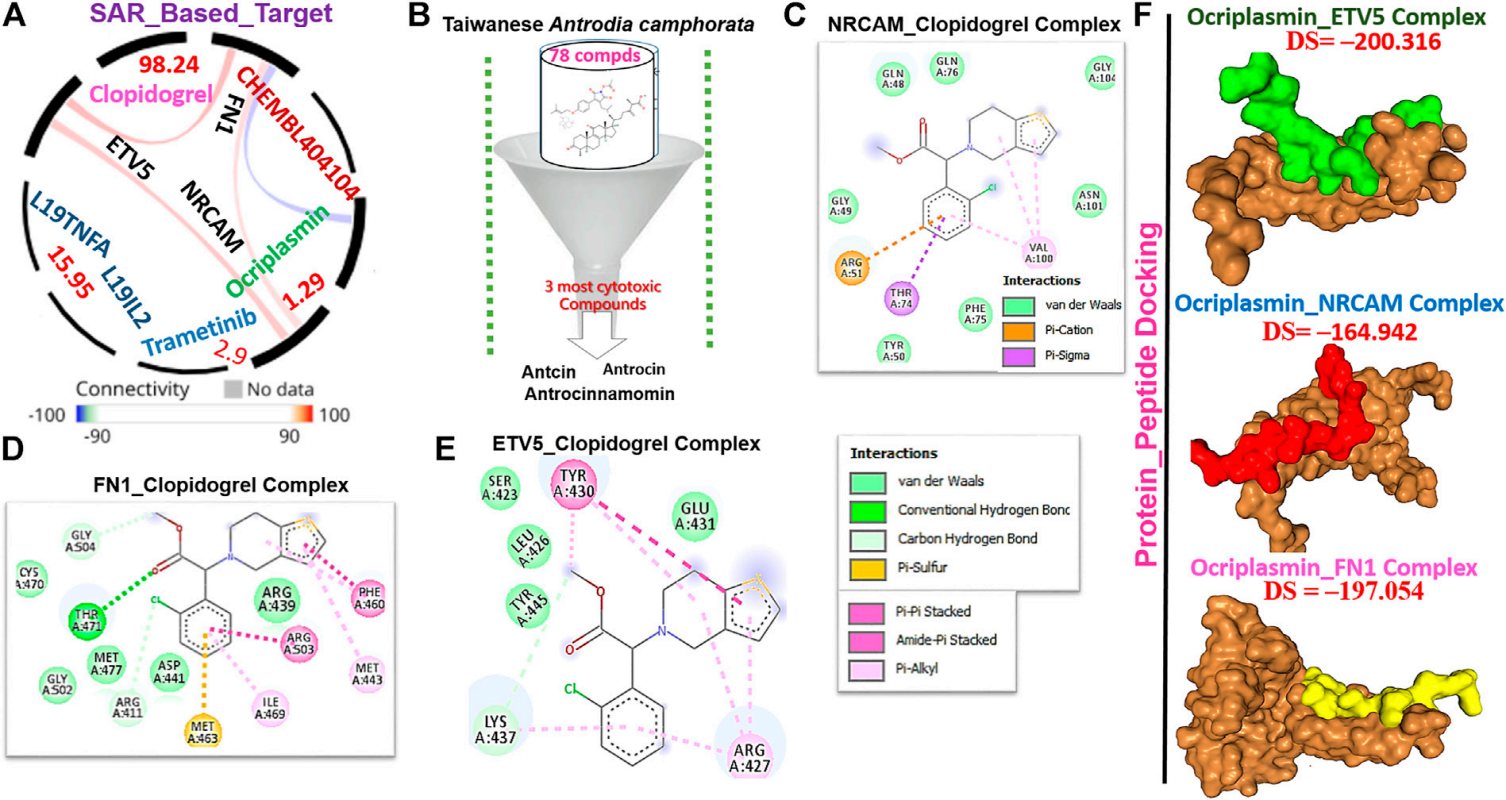
Drug target identification and molecular docking study of clopidogrel (synthetic compound) or ocriplasmin with the **t**hyroid tumor-enriched protein (TTEP). **(A)** Target connectivity mapping of FN1/ETV5/NRCAM. **(B)** Sorting and identification of potent phytocompounds from Antrodia camphorata. Molecular docking profile of clopidogrel with **(C)** FN1, **(D)** NRCAM, and **(E)** ETV5. **(F)** Protein–peptide docking of ocriplasmin with FN1/ETV5/NRCAM.

**FIGURE 10:**
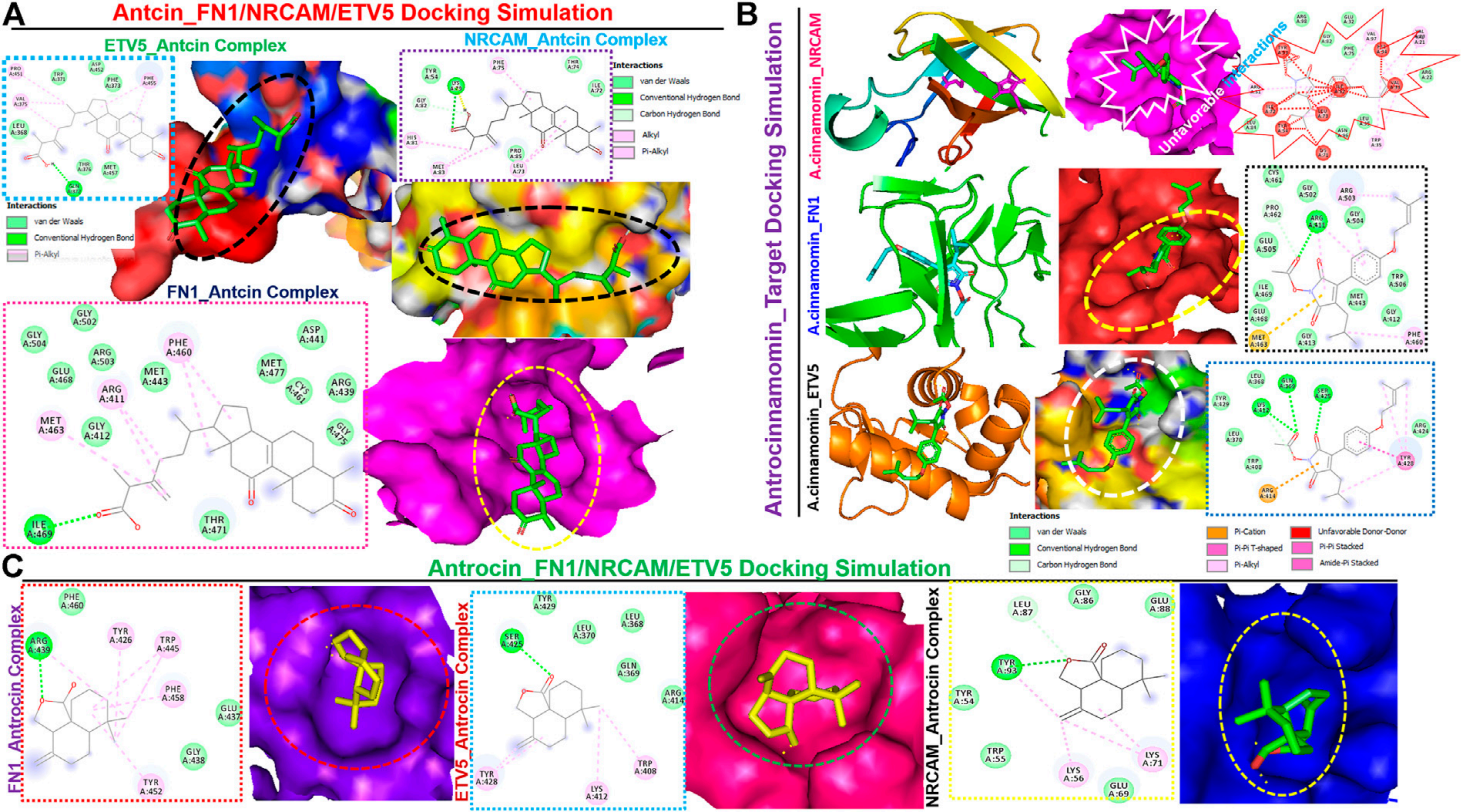
Molecular docking profile of FN1/ETV5/NRCAM with the phytocompound isolated from Taiwan *Antrodia camphorata.* Docking profile of **(A)** antcin, **(B)** antrocinnamomin, and **(C)** antrocin with FN1/ETV5/NRCAM.

We queried FN1, ETV5, and NRCAM for potential drug targets based on the structural activity relationship (SAR) and identified a synthetic compound (clopidogrel) and a peptide (ocriplasmin) with high connectivity scores and probable ligand inhibitors of the three targets (Figure 9A). Furthermore, we explored the therapeutic potential of bioactive compounds from one of the most reputable Taiwan indigenous medicinal plant (*Antrodia camphorata*). Of about 80 characterized compounds from this plant (Geethangili and Tzeng, 2011), we identified antrocinnamomin, antcin, and antrocin as the three most potent compounds primarily implicated in this plant’s bioactivity (Figure 9B). In addition, these compounds have been previously reported for anti-cancer, anti-inflammatory, and immunomodulatory activities in our laboratory and elsewhere (Chen et al., 2007; Male et al., 2008; Yeh et al., 2009; Rao et al., 2011; Yeh et al., 2013; Chiu et al., 2016; Chen et al., 2019a; Chen et al., 2019b). Our molecular docking study revealed that the synthetic compound, clopidogrel, docked well by hydrogen bonding, alkyl interaction, and several van der Waals forces to the binding cavity of FN1, ETV5, and NRCAM with binding affinities of –6.70, –5.90, and –5.50 Kcal/mol, respectively (Figures 9C–E; Table 2). On the other hand, the protein-peptide docking revealed a robust binding of ocriplasmin to ETV5, FN1, and NRCAM with affinities of ‒200.316 Pbe, ‒197.054 Pbe, and ‒164.942 Pbe, respectively (Figure 9F). Interestingly, the three phytoactive compounds (antrocinnamomin, antcin, and antrocin) demonstrated higher binding affinities (–5.50 ∼ –7.50 Kcal/mol) to FN1, ETV5, and NRCAM (Figure 10) than the affinities for ligands demonstrated by the synthetic compound and peptide (Table 2). The compounds are bonded to the active site of the ligands by hydrogen, alkyl, and pi interactions, and several van der Waals forces formed around the ligand’s backbones with several amino acid residues of the receptor’s binding sites (Figures 10A–C). Among the phytochemicals, we found that antcin exhibited higher affinities to the binding sites of all the receptors, followed by antrocinnamomin, while antrocin demonstrated the least. In addition, FN1 demonstrated high ligandability potential for the compounds, whereas NRCAM demonstrated the least (Table 2). Although antrocinnamomin docked well to NRCAM, our analysis revealed that the interactions are not favorable for ligandability (Figure 10B).

**TABLE 2:** Docking profile of TTEPs with antrocinnamomin, antcin, and antrocin isolated from Taiwan *Antrodia camphorata.*

	Clopidogrel			Antcin			Antrocinnamomin			Antrocin		
	FN1	NRCAM	ETV5	FN1	NRCAM	ETV5	FN1	NRCAM	ETV5	FN1	NRCAM	ETV5
ΔG (Kcal/mol)	−6.70	−5.50	−5.90	−7.50	−5.90	−7.0	−6.80	−5.50	−6.20	−6.80	−5.20	−5.90
Conventional	Gly504 (3.54), Arg411 (3.69), and Thr471 (2.25)		Lys437	Ile469 (2.24)	Gly82 (3.76) and	Gln372 (2.18)	Arg411 (2.21) and Pro462(3.21)	Non-Fav	Lys412 (2.96),	Arg439 (1.99)	Leu87 (3.35) and	Ser425 (2.35)
H-bond					Lys79 (3.02)				Gln369 (2.88), and		Tyr93 (1.96)	
									Ser425 (2.59)			
Alkyl interaction		Val100	Arg427 and Lys437	Met463, Arg411, and	His81	Pro451, Val375, and Phe455	Phe460 and Arg503	Non-Fav	Tyr428	Tyr426, Trp445,	Lys56 and Lys71	Trp408, Lys412, and Tyr428
				Phe460	Met83, Leu73, and					Phe458, and		
					Phe75					Tyr452		
Pi-pi stacked	Phe460		Thr430					Non-Fav	Tyr428			
Pi-sigma		Thy74										
Pi-cation		Arg51						Non-Fav	Arg414			
Pi-sulfur	Met463						Met463					
Amide-pi stacked	Arg 503											
Van der Waals forces	Cys470, Gly502, Asp441, and Arg439	Gln48, Gln76, Gly104, Asn101, Gly49, Phe75, and Thr50	Ser423, Leu426, Tyr445, and Glu431	Gly504, Gly502, Glu468, Arg503, Met443, Gly412, Met477, Asp441, Arg439, Cys461, Gly475, and Thr471	Tyr54, Pro85, Thr74, and Ile72	Trp371, Asp452, Phe373, Met457, Thr376, and Leu368	Trp506, Met443, Gly412, Gly413, Ile469, Glu468, Glu505, Pro462, Cys461, Gly502, and Gly504	Non-Fav	Tyr429, Leu370, Trp408, and Arg424	Glu437 and	Glu88, Gly86, Tyr54, Trp55, and Glu69	Tyr429, Leu370, Leu368, Gln369, and Arg414
										Gly438		

### Thyroid tumor-downregulated genes are mainly involved in the maintenance of ion and membrane structures, and the inhibition of the cell cycle at the G1/S phase transition

We also integrated differentially downregulated DEGs from the datasets and identified six downregulated DEGs, namely, MPPED2, TNFRSF11B, FHL1, CRABP1, MATN2, and TFF3, collectively known as thyroid tumor-downregulated genes (TTDGs) (Supplementary Figure S4). The expression fold change (log2) of the TTDG is shown in Table 3. Enrichment analysis revealed that the downregulated DEGs (TTDGs) are mainly involved in the maintenance of the epithelial structure, membrane depolarization, ion membrane structure-activity, and negative regulation of cell cycle G1/S phase transition (Table 4). Altogether, our analysis revealed that the TTDGs are involved in the maintenance of ion and membrane structures and inhibition of the cell cycle at the G1/S phase transition.

**TABLE 3:** Expression fold change (log2) of differentially downregulated genes (DDG) in papillary thyroid carcinoma.

	TFF3	MPPED2	TNFRSF11B	FHL1	CRABP1	MATN2
GSE3467	−3.393	−3.082	−2.941	−2.51	−2.207	−1.947
GSE3678	−4.056	−3.518	−2.391	−1.708	−3.759	−2.343
GSE5364	−2.677	−1.511	−2.371	−1.277	−2.456	−1.393
GSE9115	−5.752	−3.962	−2.779	−2.524	−5.337	−3.329
GSE29265	−3.937	−3.276	−2.158	−1.791	−1.89	−2.802
GSE29315	−2.661	−1.362	−1.136	−1.194	−1.591	−1.132
GSE33630	−4.462	−3.438	−2.505	−2.095	−3.669	−2.843
GSE35570	−5.752	−3.962	−2.779	−2.524	−5.337	−3.329
GSE58545	−6.234	−5.037	−3.87	−3.17	−4.794	−3.862
GSE60542	−3.952	−3.517	−2.478	−2.033	−3.126	−2.626

**TABLE 4:** Enriched biological processes of thyroid tumor downregulated gene (TTDG).

Index	Name	*p*-value	Adjusted *p*-value	Combined score
1	Epithelial structure maintenance (GO: 0010669)	0.005388	0.03028	1227.64
2	Maintenance of gastrointestinal epithelium (GO: 0030277)	0.005388	0.03028	1227.64
3	Regulation of membrane depolarization (GO: 0003254)	0.005388	0.03028	1227.64
4	Regulation of potassium ion transmembrane transporter activity (GO:1901016)	0.007776	0.03028	775.88
5	Ion homeostasis (GO: 0050801)	0.008967	0.03028	649.09
6	Regulation of potassium ion transport (GO: 0043266)	0.009265	0.03028	623.08
7	Negative regulation of cell cycle G1/S phase transition (GO: 1902807)	0.01046	0.03028	535.47
8	Negative regulation of G1/S transition of the mitotic cell cycle (GO: 2000134)	0.01105	0.03028	499.54
9	Positive regulation of potassium ion transport (GO: 0043268)	0.01135	0.03028	483.15
10	Regulation of ion transmembrane transporter activity (GO: 0032412)	0.01164	0.03028	467.70

## Discussion

The present study represents the largest ever transcriptomic analysis of THCA datasets. Our comprehensive, integrated analysis illuminates the potential molecular characteristics and enhances our understanding of the pathological processes of thyroid carcinoma. We integrated all the available transcriptomic datasets of the gene expression profile from THCA and non-THCA tumors and identified graded levels: grade 1 (ETV5, FN1, and NRCAM), grade 2 (ABCC3 and ETV4), grade 3 (ALDH1A3, CKS2, GALE, GDF15, and GGCT), and grade 4 (MAMLD1, MAP4K4, PSD3, PTP4A3, TNFRSF10B, TNIK, TUSC3, and ZMAT3) levels of biomarker signatures for THCA (TTEPs), and two microRNAs collectively known as thyroid tumor-enriched miRs (TTEmiRs). We found that TTEPs are associated with tumor stages, poor surgical outcome, distance metastasis, and worse prognosis in the cohorts. In addition, TTEPs demonstrated a cooperative association with the T-cell exclusion mechanism of immune-invasive phenotypes of THCA and induced significant pathological interactions *via* EMT, cell-matrix adhesion mechanism, and transcriptional dysregulation in PTC.

Our differential gene expression analysis based on TCGA cohorts of THCA revealed that all members of the TTEPs are highly significantly (*p* < 0.001) overexpressed in the thyroid tumor tissue compared to the adjacent normal tissue. The highly significant levels of overexpression of these genes strongly validated our large-scale transcriptomic data analysis and suggested that these signatures may be a useful genetic biomarker for diagnosing PTC. DNA methylation is a crucial epigenetic modulation in mammalian genomes and plays a crucial role in regulating gene expression and may serve as a noninvasive biomarker for cancer diagnosis and prognosis (Robertson, 2005; Lawal et al., 2021a). Compared with the normal tissue, the significant hypomethylation of the TTEPs in the PTC tumor tissue is in sharp contrast with the expression levels of these genes in the corresponding tumor sample. These negative associations between the mRNA level and methylation status of the TTEPs suggest the epigenetic regulation of the TTEPs, which could be attributed to the recruitment of the methyl moieties containing gene repressor proteins or by inhibiting the binding of transcription factors to DNA (Robertson, 2005; Shinawi et al., 2013).

Cancer cell interactions with the TME play an important cancer etiologic role in various tumors *via* distinct mechanisms involving a phenotype of dysfunctional T-cells and a phenotype of T-cell exclusion (Chen et al., 2021a; Wu et al., 2021). Our analysis revealed that all members of TTEPs demonstrated a cooperative relationship with the infiltrating levels of different categories of the cells of immunosuppressive and immune response in THCA. Infiltrations of these cells within the tumor vary with the immune status of the host and have pertinent prognostic roles. CAF, Tregs, TAM, and MSDC are immunosuppressive cells that inhibit the activities of CTL causing the exclusion of T cells and the induction of invasive phenotypes (Chen et al., 2021a). However, some tumors have abundant infiltration of immune cells; these cells are dysfunctional and unable to activate the immune response, a condition known as T-cell allergy (Lawal et al., 2021a; Lawal et al., 2021d). We found that the mRNA expression levels of the TTEP show no significant (*p* > 0.05) association with the infiltration levels of cytotoxic lymphocytes (B cells, CD4 naïve, CD4 T, CD8 naïve, CD8 T, MAIT cells, NK cells, and γδ T cells), and negative association (*p* < 0.05) with infiltration of T-helper cells and neutrophils. However, the mRNA expression levels of the TTEP show a significant and positive (*p* < 0.05) association with the infiltration levels of immunosuppressive cells (CAF, MDSC, TAM, nTreg, iTreg, and Tr1), dendritic cells, macrophages, and monocytes. These immunosuppressive phenotypes are markers of T-cell exclusion. Hence, our results strongly suggested that TTEPs exhibited a cooperative association with the T-cell exclusion mechanism of immune-invasive phenotypes in PTC.

During innate and adaptive immune responses, the TME secretes growth factors that induce EMT, one of the hallmarks of cancer (Kalluri and Weinberg, 2009; Mittal, 2018). EMT mediated the interruption of cellular polarity and cell–cell adhesion, cell-matrix adhesion, and cytoskeleton remodeling (Roche, 2018). In addition, EMT induced marked resistance to therapy and apoptotic stimuli (Mittal, 2018). However, more knowledge about the deregulatory mechanism of EMT in THCA, its control, and EMT-THCA-specific molecular markers is necessary. Indeed, all our enrichment and functional analyses revealed that the TTEPs are strictly involved in transcriptional regulation of EMT-associated processes, including the ABC transporters, interactions of ECM-receptors, proteoglycans in malignance, focal adhesion, actin cytoskeleton regulation, and cell adhesion molecules.

Consistently, our analyses revealed that patients with altered TTEP profiles showed little response to surgical intervention, increased incidence of distant metastases, tumor recurrence, and significantly lower survival. These findings all suggest that the TTEPs represent a specific biomarker set for assessing thyroid tumor staging, metastasis, prognosis, and therapeutic response.

An important challenge in cancer treatment is the development of resistance to chemotherapeutic drugs (Choi et al., 2020). While some cancer patients may achieve an initial remission with present therapeutic regimens, tumor recurrence is observed in most patients even with aggressive treatment (Steinbach et al., 2002). Our analysis revealed that with the exclusion of PTP4A3 and GGCT, the expression levels of the TTEPS were significantly (*p* < 0.05) associated with the resistance against several classes of small-molecule drugs. On the contrary, we observed that these gene signatures induced increased sensitivity of cancer cell lines to several MEK inhibitors, including trametinib, selumetinib, 17-AAG, RDEA119, PD-0325901, (5Z)-7-oxozeaenol, and, a mutant-BRAF kinase inhibitor, dabrafenib (Figure 7), suggesting that this class of anti-cancer drugs may still be useful in the treatment of thyroid cancer.

A previous study reported that hypomethylation induced the overexpression of miR-146b and miR-21 in THCA with consequent conferment of proliferative and invasive tumor phenotypes (Ortiz et al., 2018), in agreement with our findings. He et al. (2005) also identified the upregulation of miRNA 146b in 20 patients with PTC (He et al., 2005). Similarly, Geraldo et al. (2012) reported that miR-146b-5p is highly expressed in PTC and is considered an important diagnostic biomarker for this cancer type. It has been reported that miR-146b-5p plays an oncogenic role by putatively binding to the 3′ UTR of the targets to regulate the TGF-β signaling pathway in favor of thyroid cancer tumorigenesis (Geraldo et al., 2012). Similarly, our validation of the miRs using TCGA cohorts’ dataset revealed that miR-21-5p and miR-146b-5p are more than 100-fold overexpressed in THCA compared to the non-THCA cohorts. Therefore, the expression levels of these miRs can be used as reliable non-coding biomarkers of thyroid cancer. However, our dataset analysis revealed no significant differential expression of these miRs among the different THCA histology, suggesting that miR-21-5p and miR-146b-5p are generally thyroid tumor-specific rather than histologically-specific. Indeed, several experimental studies have identified these two miRs as important regulators of different thyroid tumor histology including ATC, PTC, and FTC (Zhang et al., 2013; Mancikova et al., 2015; Hu et al., 2016). However, miR-146b-5p expression has been identified to increase significantly throughout stages I to III of PTC (Zhang et al., 2013), suggesting its involvement in thyroid tumor progression. Our functional analyses of miR-146b-5p and miR-21-5p revealed their involvement in various cellular functions, including immune response, EMT, cell–cell adhesion, cell growth, and stemness. These findings are supported by various pieces of experimental evidence implicating these miRs in migration, proliferation, invasion, colony-forming ability, cell cycle, and chemotherapy resistance (Gonda et al., 2010; Chou et al., 2013; Deng et al., 2015; Ortiz et al., 2018). Furthermore, hsa-miR-146b-5p expression has been regarded as an independent risk factor for poor prognosis in PTC together with lymph node metastasis and advanced tumor stage (Chou et al., 2013).

Interestingly, we identified a synthetic compound (clopidogrel) and a peptide (ocriplasmin) as the most probable ligand inhibitors of the TTEPs. Natural products serve as an important reservoir of bioactive metabolites for the treatment of several diseases (Lawal et al., 2015; Lawal et al., 2016; Onikanni et al., 2021). Our molecular docking study revealed that antrocinnamomin, antcin, and antrocin, the bioactive compounds from one of the most reputable Taiwan indigenous medicinal plant (*Antrodia camphorata* **),** demonstrated higher binding affinities (–5.50 ∼ –7.50 Kcal/mol) to FN1, ETV5, and NRCAM (Figure 10) than the affinities for ligands demonstrated by the synthetic compound and peptide (Table 2). The phytocompounds are bound to the active site of the ligands by hydrogen, alkyl, and pi interactions and several van der Waals forces formed around the backbones of the ligands with numerous AAs residues of the receptor’s binding sites (Figures 10A–C). Among the phytocompounds, we found that antcin exhibited higher affinities to the binding sites of all the receptors, followed by antrocinnamomin, while antrocin demonstrated the least. In addition, FN1 demonstrated high ligandability potential for the compounds whereas NRCAM demonstrated the least (Table 2). Although antrocinnamomin docked well to NRCAM, our analysis revealed that the interactions are not favorable for ligandability (Figure 10A).

Collectively, by the integration of various GEO datasets, we not only identified various significant coding and non-coding theranostic biomarkers that underlay PTC progression but also found graded levels of the hub genes, which may be considered novel and potential biomarkers of thyroid cancer. Cohorts from TCGA database were finally explored to affirm the precision of our findings and we found that the identified biomarkers were associated with tumor stages, poor surgical outcome, distance metastasis, immune-invasive phenotypes, and worse prognosis of the cohorts. Therefore, our results provide novel and reliable biomarkers for elucidating the pathogenesis of thyroid cancer, which will be helpful in future clinical applications in prognosis, diagnosis, target therapy, and monitoring treatment outcome in thyroid cancer.

Furthermore, we identified MPPED2/TNFRSF11B/FHL1/CRABP1/MATN2/TFF3 as important downregulated DEGs in PTC. MPPED2 has been reported to act as a tumor suppressor that inhibits PTC *via* activation of PTEN and suppression of the AKT pathway (Li et al., 2021). Qu et al. (2016) also reported the downregulation of TNFRSF11B in PTC. Our study, therefore, serves as a validation for the previous studies where MPPED2, TNFRSF11B, FHL1, CRABP1, or MATN2/TFF3 were individually or jointly found to be downregulated in thyroid cancer (Barros-Filho et al., 2015; Gomez-Rueda et al., 2016; Qu et al., 2016; Sepe et al., 2018; Li et al., 2021). Interestingly, we found that these gene signatures are mainly involved in the maintenance of ion and membrane structures and the inhibition of the cell cycle at the G1/S phase transition. This stresses the pathological importance of ion and membrane structure homeostasis and cell cycle at the G1/S phase transition in the development of papillary thyroid carcinoma.

However, the limitation of our study must be acknowledged. The absence of patient cohorts to corroborate our findings marked a critical area that required further studies. Moreover, functional studies, including potential target validation and the therapeutic potential of antcin in cells and tumor-bearing mice are necessary for the clinical applicability of our findings.

## Conclusion

Collectively, our analysis of large-scale transcriptomic datasets of THCA identified graded levels of gene and microRNA biomarkers, implicating the underlying mechanisms involving T-cell exclusion, EMT, and cell-matrix remodeling. Further investigation is warranted for future clinical applications.

## Data Availability

The original contributions presented in the study are included in the article/Supplementary Material; further inquiries can be directed to the corresponding author.
