# Supplementary material for: Large-scale transcriptomic analysis of coding and non-coding pathological biomarkers, associated with the tumor immune microenvironment of thyroid cancer and potential target therapy exploration
Source: Front Cell Dev Biol. 2022 Aug 3;10:923503. doi: 10.3389/fcell.2022.923503 (PMC9384576; doi:10.3389/fcell.2022.923503)
Supplement: Supplementary file 1 [file DataSheet1.docx]

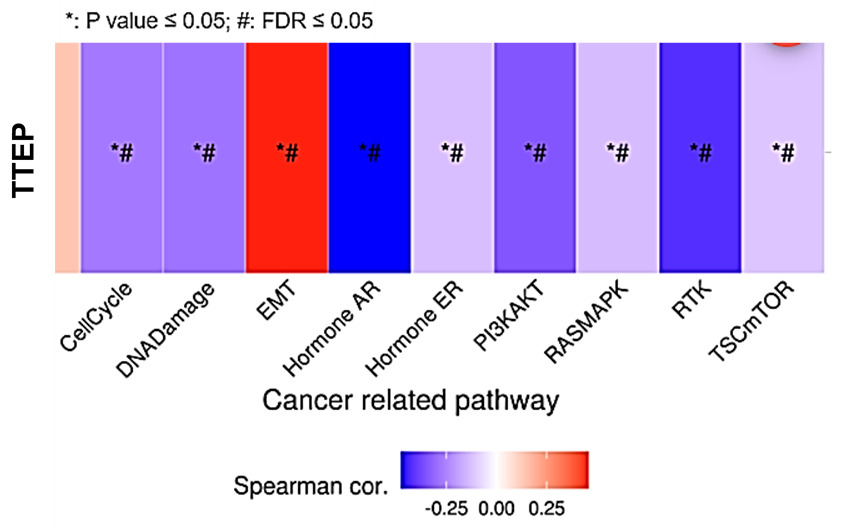


**Figure S1:** Heatmap plot of the association between the expression levels of the TTEPs and the immune infiltration in tumors of the TCGA-PTC cohorts. The red and blue plots represent positive and negative associations between the mRNA expression and pathways/pathways at FDR adjusted p-value of 0.05.


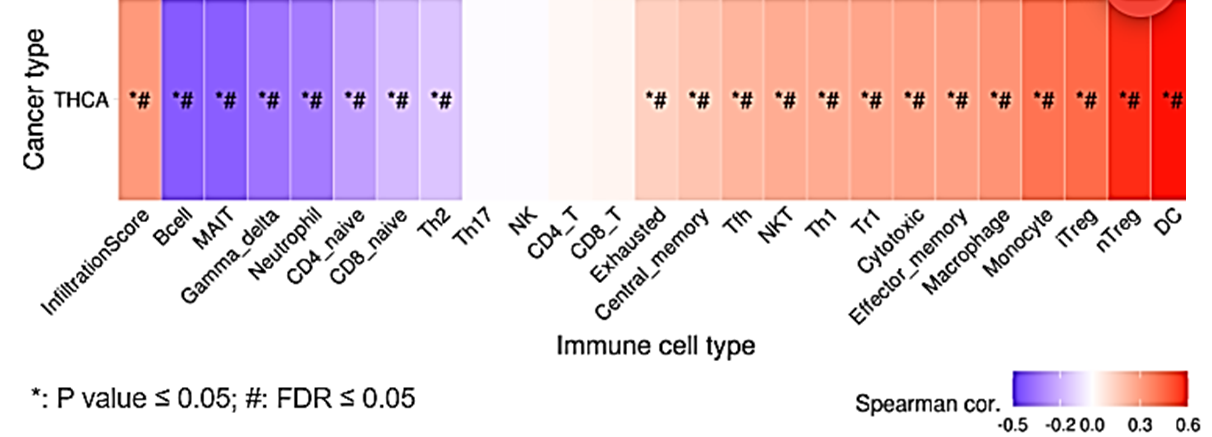


**Figure S2:** Heatmap plot of the association between the expression levels of the TTEPs and the activation of major cancer-associated pathways. The red and blue plots represent positive and negative associations between the mRNA expression and pathways/pathways at FDR adjusted p-value of 0.05.


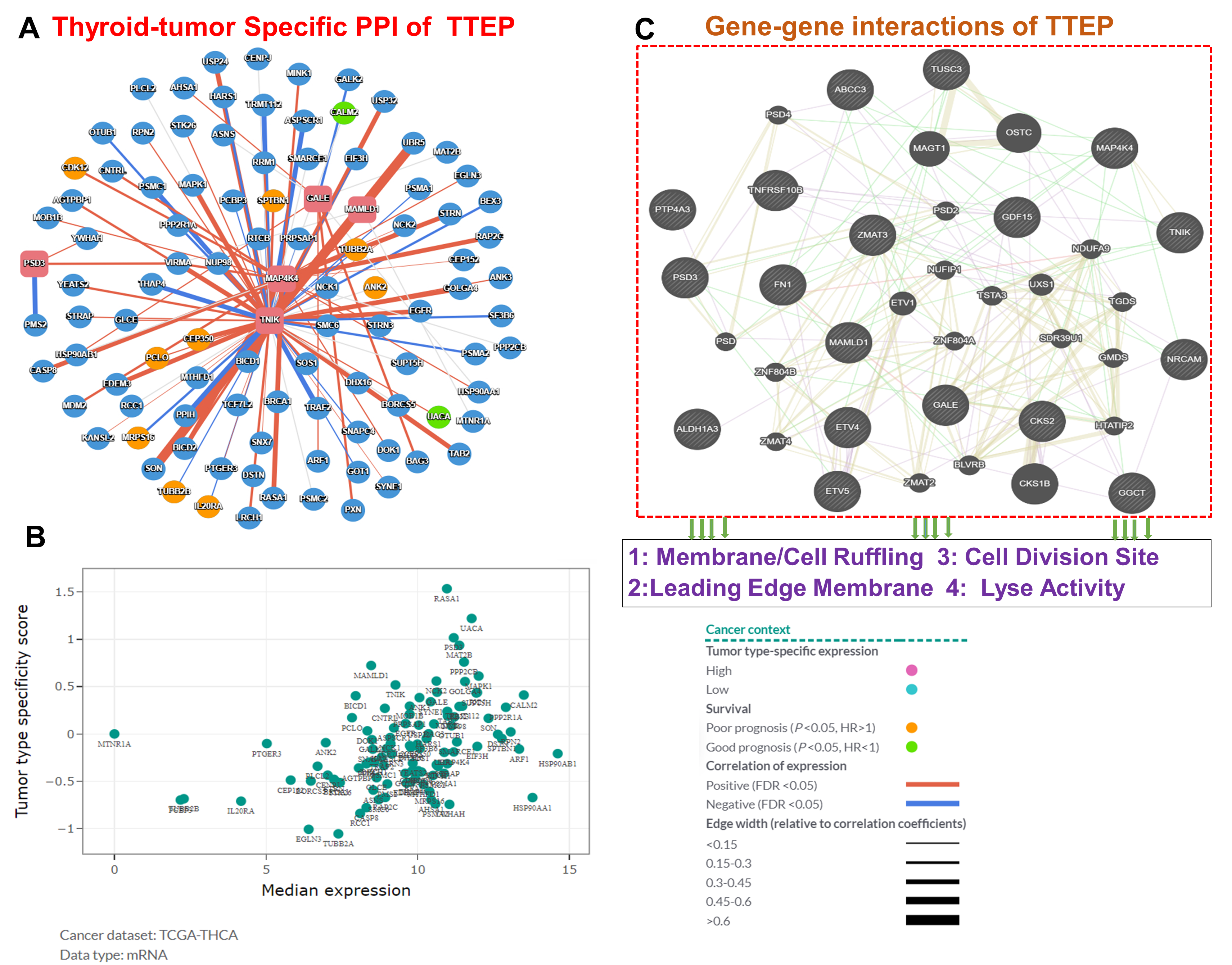


**Figure S3. N**etwork plots of (A) thyroid tumor-specific protein-protein interactions of the DEGs, (B) DEGs mediated Tumor type specific prognostic proteins (C) gene-gene interactions of TTEPs. the thyroid-specific PPI was queried based on the thyroid cancer mediated interactions of the protein and its consequence on the oncogenic and prognostic role of other proteins in the thyroid cancer tumor DEGs associated Tumor type specific proteins


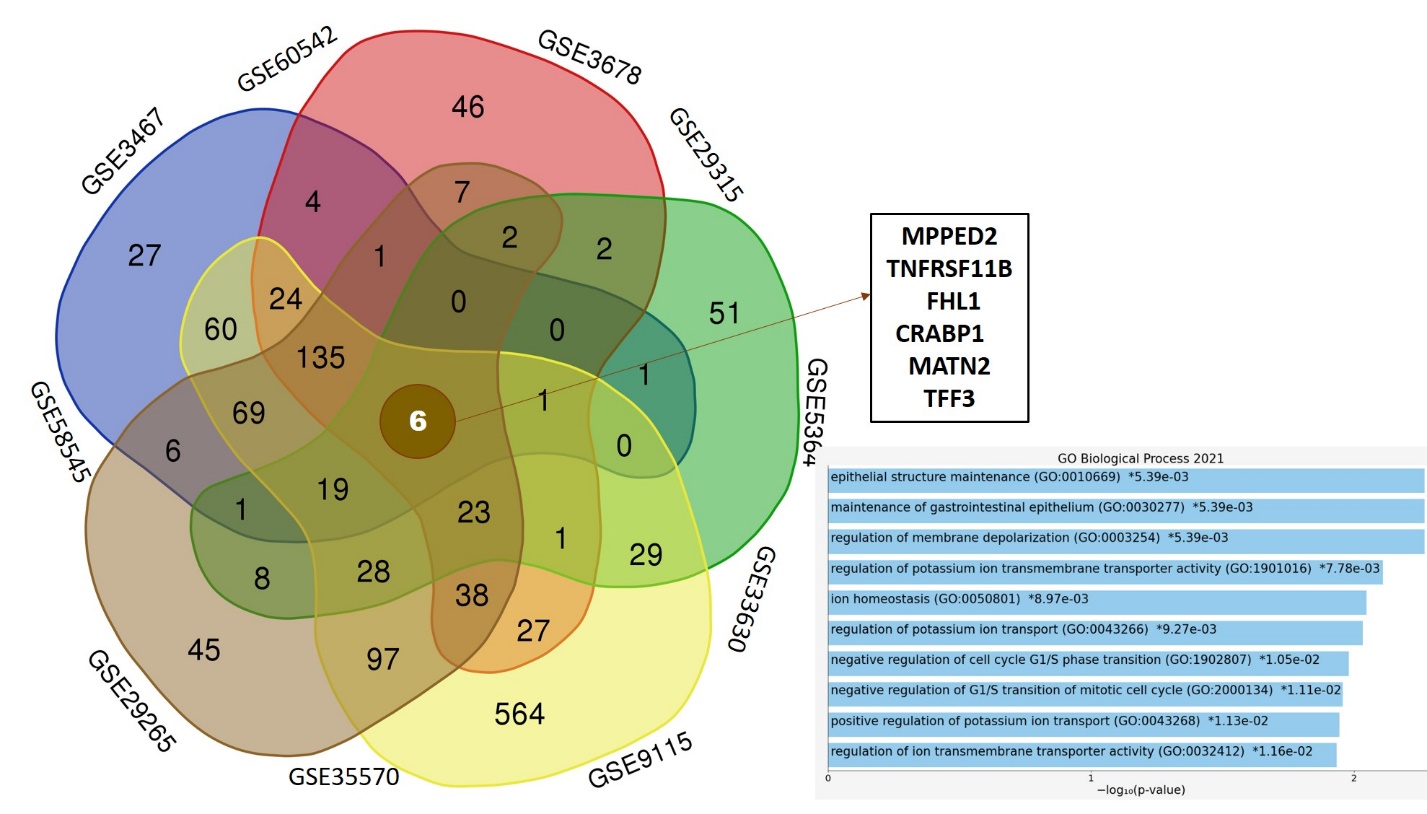
**Figure S4:** Venn diagram and enrichment plot of the differentially downregulated genes (TTDG) in THCA datasets.
